# Mechanical valve replacement for patients with rheumatic heart disease: the reality of INR control in Africa and beyond

**DOI:** 10.3389/fcvm.2024.1347838

**Published:** 2024-02-09

**Authors:** Peter Zilla, Paul Human, Tim Pennel

**Affiliations:** Christiaan Barnard Division of Cardiothoracic Surgery, University of Cape Town and Groote Schuur Hospital, Cape Town, South Africa

**Keywords:** rheumatic heart disease, international normalised ratio (INR), mechanical heart valve (MHV), low- to middle-income countries (LMICs), anticoagulation (AC)

## Abstract

The majority of patients requiring heart valve replacement in low- to middle-income countries (LMICs) need it for rheumatic heart disease (RHD). While the young age of such patients largely prescribes replacement with mechanical prostheses, reliable anticoagulation management is often unattainable under the prevailing socioeconomic circumstances. Cases of patients with clotted valves presenting for emergency surgery as a consequence of poor adherence to anticoagulation control are frequent. The operative mortality rates of reoperations for thrombosed mechanical valves are several times higher than those for tissue valves, and long-term results are also disappointing. Under-anticoagulation prevails in these regions that has clearly been linked to poor international normalised ratio (INR) monitoring. In industrialised countries, safe anticoagulation is defined as >60%–70% of the time in the therapeutic range (TTR). In LMICs, the TTR has been found to be in the range of twenty to forty percent. In this study, we analysed >20,000 INR test results of 552 consecutive patients receiving a mechanical valve for RHD. Only 27% of these test results were in the therapeutic range, with the vast majority (61%) being sub-therapeutic. Interestingly, the post-operative frequency of INR tests of one every 3–4 weeks in year 1 had dropped to less than 1 per year by year 7. LMICs need to use clinical judgement and assess the probability of insufficient INR monitoring prior to uncritically applying Western guidelines predominantly based on chronological age. The process of identification of high-risk subgroups in terms of non-adherence to anticoagulation control should take into account both the adherence history of >50% of patients with RHD who were in chronic atrial fibrillation prior to surgery as well as geographic and socioeconomic circumstances.

## Introduction

In industrialised countries with predominantly degenerative heart valve pathologies, the proportion of patients receiving a mechanical prosthesis has steadily decreased to just 10% of all valve replacements (1). The situation is distinctly different in low- to middle-income countries (LMICs), where RHD still prevails. Contrary to general perceptions, the rate of prevalence of RHD has been increasing steadily, reaching 41 million in 2019 (2, 3). Since 2017, the number of deaths from RHD has also been increasing (2). In a global context, RHD remains the most common cause of death from valvular heart disease, with an incidence rate almost double that of non-rheumatic valve lesions (2, 4).

As patients with RHD are on average 30 years younger than their Western counterparts with degenerative valve diseases (5), mechanical prostheses are favoured whenever a minimum level of anticoagulation compliance can be expected and when valves cannot be repaired (6, 7).

Unfortunately, for the vast majority of patients, their socio-economic circumstances are not conducive for availing anticoagulation therapy. Yet, in a resource-deprived environment where cardiac surgical capacity is insufficient (8, 9), the fear of re-operations often leads to an inordinate preference for mechanical valves. Hence, LMICs tend to adhere more rigidly to Western age guidelines (5, 10) than industrialised countries, where the age bracket for tissue valves has continuously been downward-adjusted in spite of their superior monitoring abilities (11, 12). This strict choice of mechanical valves over tissue valves results in a situation where the vast majority of redo valve surgeries in LMICs are done for valve thrombosis of mechanical prostheses (13) and not for the degeneration of tissue valves.

The fact that these clotted valves are not a rare phenomenon but occur disturbingly often, is not only an observation of clinicians on the ground (13), but also reflected in the poor survival results of these patients (14–18) and often directly related to poor anticoagulation compliance (16, 19–24). Nonetheless, unwavering optimism often prevails in clinicians, with the belief that patients will somehow manage their anticoagulation. Underlying this firm position is the belief that the need for a re-operation of a failed tissue valve represents the worst of all outcomes. Nothing confirms this immutable optimism that the vast majority of patients will cope with anticoagulation better than the fact that patients with thrombosed valves often receive mechanical valves again, although they keep returning for redo valve surgeries (13). Indeed, 19% of patients needing redo surgeries for clotted mechanical valves were shown to have at least another re-operation when the second mechanical valve clotted again (13).

Therefore, when it comes to heart valve choices in LMICs, we tend to follow a clinical decision-making process that is based on Western assumptions rather than the critical consideration of local realities. In a major study across Africa, it was found that approximately 90% of patients received mechanical valves and, although every 10th patient stopped warfarin, 12% had no international normalised ratio (INR) monitoring at all, while 34% had only sporadic INR measurements (25).

### Outcomes after mechanical valve replacement in patients with RHD in LMICS

Valve thrombosis is the predominant major valve-related event (13) in regions with poor INR control, often requiring emergency redo valve surgeries within less than 4 years after the initial surgery (13). As a consequence of the clinical emergency presentation, the re-operative mortality rate is high (14). Since a significant proportion of valve thromboses events are likely to occur in remote areas, deaths may often not be recognised as “valve-related.”

As such, long-term results may, in reality, be worse than some local studies with selected patients may suggest. For instance, a third of all late deaths fell into this category in a recent study from Uganda (26). In a previous follow-up study at our institution, only 21% of remote deaths could be clearly assigned to “valve thrombosis”; however, if one added other categories of “sudden death” or “congestive cardiac failure with pulmonary edema,” that result rose to 57%. In an Ethiopian study with a 27% 6-year mortality rate for single mechanical MVR, 22% of deaths were “sudden” or “unknown,” 7% were lost to follow-up, and 44% died of “heart failure” without excluding underlying valve thrombosis (16). As such, the 6-year mortality rate after mechanical valve replacement of 21% in Cameroon (16) may also have been too optimistic. Confirming this, the 10-year death rate in young patients in India was 41% after mechanical MVR and 28% after mechanical AVR (18). However, for RHD, mechanical valve replacement showed poor results even if the patients underwent surgery in an industrialised country. In Australia's aboriginal population, the 10-year mortality rate after mechanical valve replacement was 38% (27). Similarly, Maori and Pacific Island women returning from mechanical heart valve replacement in New Zealand had a 7–8-fold higher relative risk of death compared with their European counterparts operated at the same institution (17) and more than double the risk of dying compared with those with tissue valves in spite of a 2.8-fold higher risk of re-operation in the latter group. In young adults in Saudi Arabia who underwent double valve replacement for RHD, the 15-year survival rate for those with bioprosthetic valves was 92% compared with 76% for those with mechanical valves (28), and, in a locally operated series in the Fijis, the 10-year mortality rate for mechanical heart valves for those with RHD was 24%, with death occurring on average 3.2 years after surgery (15).

### Insufficient compliance with anticoagulation

Without trivialising the seriousness of bleeding complications associated with over-anticoagulation, under-anticoagulation in LMICs by far exceeds the former (20, 29–34). Catastrophic clinical emergencies due to clotted valves have been linked to sub-therapeutic INR ranges falling below the recommended 2.5–3.5 (20, 35). In two African studies involving patients with RHD who had undergone mechanical valve replacement, most thromboembolic complications (20), including clotting of the valves (35), occurred at an INR <2.

Furthermore, under-anticoagulation has been linked to poor INR monitoring. For each 10% of missed INR tests, the odds of under-coagulation increase by 14% (36). Since LMIC patients require surgery predominantly for RHD (8, 9, 37), under-adherence is aggravated by the fact that 40% of patients with RHD undergoing valve surgery are already in atrial fibrillation (AF) pre-operatively and one-third of the remaining 60% develop AF after surgery (38).

While anticoagulation control is also often suboptimal in industrialised countries (36, 39), poor adherence is a hallmark of LMICs. In an Ethiopian cross-sectional study, it was found that patients spent 52% of their time in sub-therapeutic INR ranges (30). In the Fijis, 39% of patients with mechanical valves either had no or poor adherence (21), and in an Indian study, it was found that only 8% were fully compliant (33) compared with 25% in South Africa (40).

**Figure 1:**
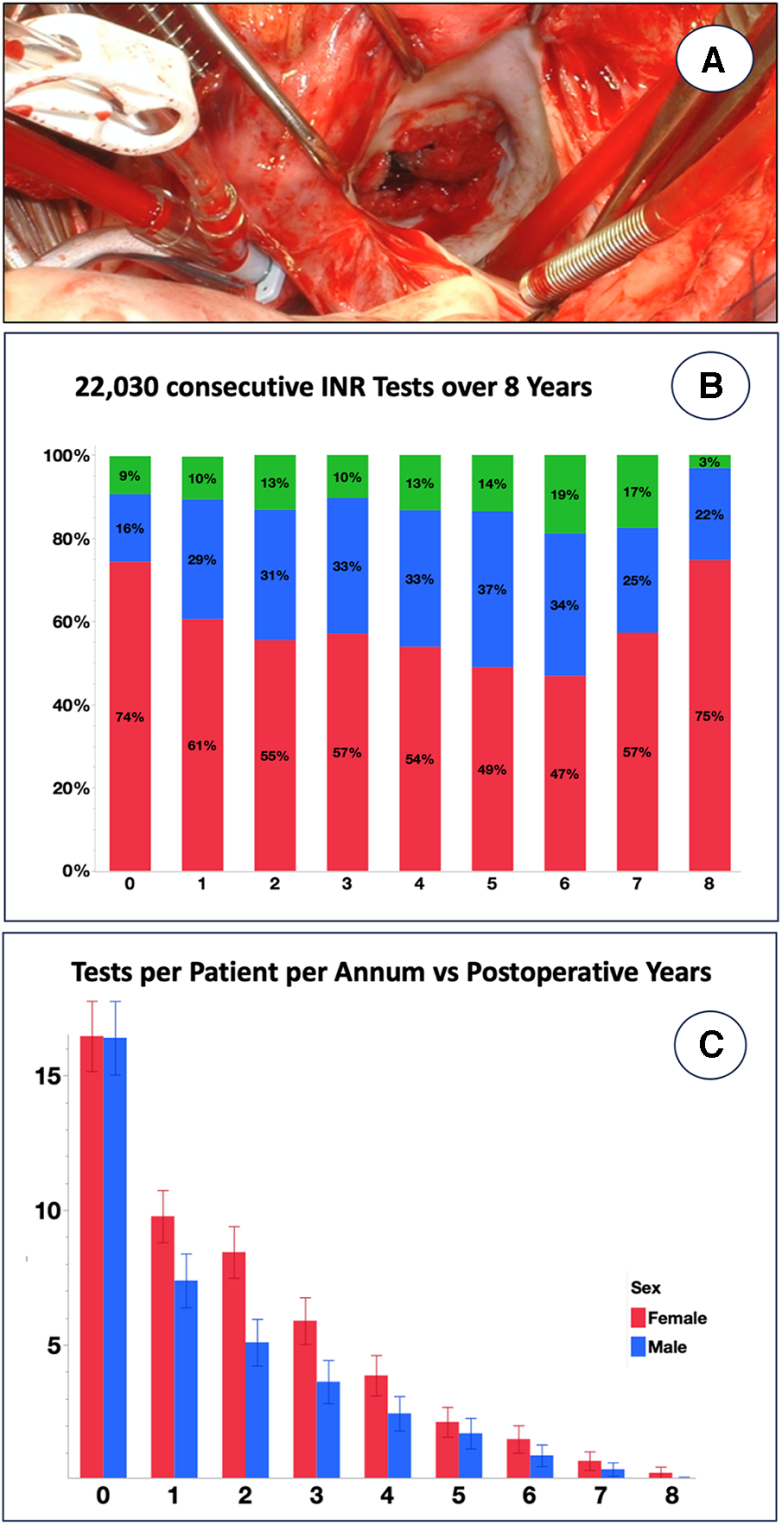
Eight-year INR follow-up of 552 consecutive patients receiving a mechanical prosthesis for rheumatic heart disease at Groote Schuur Hospital, University of Cape Town between 2015 and 2023. (**A**) Emergency re-replacement of clotted mechanical mitral valve <4 years after primary operation in a patient with notoriously low INR results [from (5) with permission]. (**B**) A total of 21,826 INR tests were recorded on the National Health Laboratory Database regardless of where the test was done. Although fluctuating over the years, INRs were below range most of the time [the percentage of INR time in range (TTR%) was 27.2% ± 24.1% (blue); below range was 61.4% ± 30.5% (red), and above range was 11.4% ± 17.3% (green)]. (**C**) Frequency of INR tests per patient, shown separately for men and women, was one every 3–4 weeks on average in the first post-operative year, but it fell progressively to 6–7/year at year 2 and eventually had fallen below 1 year by year 7.

The degree of non-compliance becomes particularly evident when non-adherence is expressed as the percentage of time within the therapeutic INR range (%TTR). In industrialised countries, “safe anticoagulation” is defined as a TTR ≥60%–70% (39, 41), *de facto* lying between 59% and 67% according to a meta-analysis of 38 studies (32). Time below range and thromboembolism exhibits a significant correlation (32). Reflecting a dangerously low compliance level, the TTR has been found to be notoriously low in LMICs, e.g., 43% in China (29), 42% in Thailand (31), 49% in South Africa (20), 28% in Ethiopia (30), and 44% in India (33), and <40% in a multi-country LMIC study (34). Most of the time, INRs were sub-therapeutic (30). Our own data obtained at the University of Cape Town confirm this trend, in spite of the fact that the drainage area is one of the best-administered provinces in sub-Saharan Africa, with two-thirds of the population living in a metropolis with access to three teaching hospitals offering open heart surgery. Of 21,826 tests conducted over 8 years in 552 consecutive mechanical heart valve recipients, the results revealed that only 27% of therapeutic time fell within the therapeutic range overall (Figure 1B). Over a period of 7 years, the frequency of post-operative tests decreased from 1 every 3–4 weeks to less than 1 per year (Figure 1C).

### Risk-factors for poor compliance

Risk factors for under-adherence are young patient age (21, 40, 42, 43), lack of formal education (42), which is indirectly also associated with unemployment (21),and female gender (5, 21, 44). Unfortunately, some of these patient characteristics are, at the same time, the hallmark features of RHD, for which a significant association with low socio-economic circumstances exists (25, 37). Accordingly, a South African anticoagulation study in patients with RHD found those patients who completely defaulted on their INR controls to be significantly younger than those in the compliant group (35 vs. 43 years) (5). The observation that young patient age generally leads to lower adherence was highlighted by the fact that it also applied to patients who did follow-up with their INR testing but did so irregularly (40, 42).

Gender-wise, an overall prevalence of the female gender was continually shown (5, 44), confirming the findings of Thomson–Mangnall's key study from the South Pacific (21). However, apart from young age and female gender being associated with non-adherence to warfarin therapy, other independent predictors of discontinuation of warfarin therapy were related to those who did not understand why warfarin was needed in the first place, had a history of forgetting to take warfarin and had a travel time to a heart clinic exceeding 1 h (21). The last-mentioned predictor can be assumed for a majority of patients in low-income countries. There also seems to be an individual metabolic component. Counterintuitively, patients with a lower percentage of time in the therapeutic range typically had more INR tests done per year than those with good INR control, indicating an individual preponderance to wider INR fluctuations (40).

Our analysis during the initial 30 post-operative days confirmed this observation: Applying partition modelling of the INR test intervals (INR gaps) against therapeutic INR ranges showed that the percentage of time in the therapeutic range was the highest when the INR gap was ≥17 days, less so when the interval fell between 4 and 17 days, and worse when it fell below 4 days. While this provisional criterion for fewer INR tests may allow a narrowing of the focus group, the low overall number of tests “in therapeutic range” shows that, in a majority of patients, sub-optimal anticoagulation coincides with dismal surveillance. To what extent poor surveillance overlaps with poor medication compliance remains speculative—a topic in which anticoagulation and anti-retroviral therapy share some ground. Yet, cultural contributors also play a strong role. Although the time in therapeutic range was indistinct between South African black, white, and mixed-race women in our cohort, it was distinctly lower in black men vs. white (*p* = 0.015) and mixed-race (*p* = 0.016) men.

### Balancing harm

Most importantly, the risks in the use of mechanical valves in regions dominated by RHD are acute, and often catastrophic, events. In an LMIC such as South Africa, 74% of redo valve operations were for clotted valves less than 4 years after the original operation, with 73% presenting as clinical emergencies (44). However, because of the acute nature of the event, re-operations for mechanical valves are associated with significantly high mortality rates. In a study from Turkey, it was found that redo surgery for mechanical valves exhibited a three times higher operative mortality rate than that for tissue valves (14), and 16% of South African patients experienced critical post-operative complications following the re-operation of a mechanical valve (13). In a cohort of the 1990s involving Maori and Pacific Island women, it was found that serious thromboembolic events occurred in 57% of women with mechanical valves within 10 years. Although these first-generation tissue valves used in these studies experienced a 3-fold higher re-operation rate than mechanical valves, the relative risk of death was 2.2 times higher after mechanical valve replacement (17). In a most recent study of pregnant women among Bangladeshi patients with mechanical valves, it was found that 12% had thrombus formation on the leaflets and 3% had warfarin embryopathies, with 35% requiring termination of pregnancy in the first trimester (45).

When considering tissue valves in young patients with RHD, circumstances have to be taken into account that hold different levels of significance in Western countries. A delayed re-intervention, or even the lack of it, with the associated ventricular damage and increased mortality (46), is predominantly seen in regions where even primary operations are provided to only a fraction of those in need (8, 9, 47).

At the same time, mortality of tissue valve re-operations has continually decreased over time. While it was over 40% in the 1960s/1970s (48), the operative mortality rate of a first re-operation in a modern series is 3%–4% for AVRs (49–51) and 4%–8% for MVRs (52, 53).

As much as valve-in-valve TAVRs are seen as a remedy to further lower the bar towards tissue valves in industrialised countries, they will remain a distant dream until costs have significantly decreased and delivery has been simplified to reflect the largely unsophisticated infrastructure of LMICs outside metropolitan centres where the majority of patients with RHD reside (8, 9).

### Unmet needs

The dilemmas faced by those who need heart valve replacements for RHD in LMICs are 2-fold: first, contemporary heart valves are catering for the elderly patients of industrialised countries, where mechanical prostheses were nearly abandoned in favour of tissue valves. As such, there is minimal commercial incentive to produce less thrombogenic valve designs. At the same time, the increasing number of older patients receiving tissue valves contributed to the limited translation of the exciting scientific breakthroughs of new tissue treatments over the past decades. The slower bioprosthetic degeneration processes observed in older patients allowed commercial valve manufacturers to stall implementing the often radically different tissue treatments (54, 55), thus avoiding the costly regulatory processes of non-incremental changes. Yet, there may be hope on the horizon for the hitherto neglected millions of young heart valve recipients for RHD, as their plight is shared with those patients of industrialised countries who are too young for contemporary TAVR and are therefore excluded from transcatheter solutions.

With the hopes for more reliable anticoagulation options dashed in LMICs, when direct oral anticoagulants (DOACs) were not approved for mechanical heart valves, the only alternatives are either significantly less thrombogenic mechanical prostheses or radically different materials for soft-leaflet valves that make them as durable as well-anticoagulated mechanical valves.

**Figure 2:**
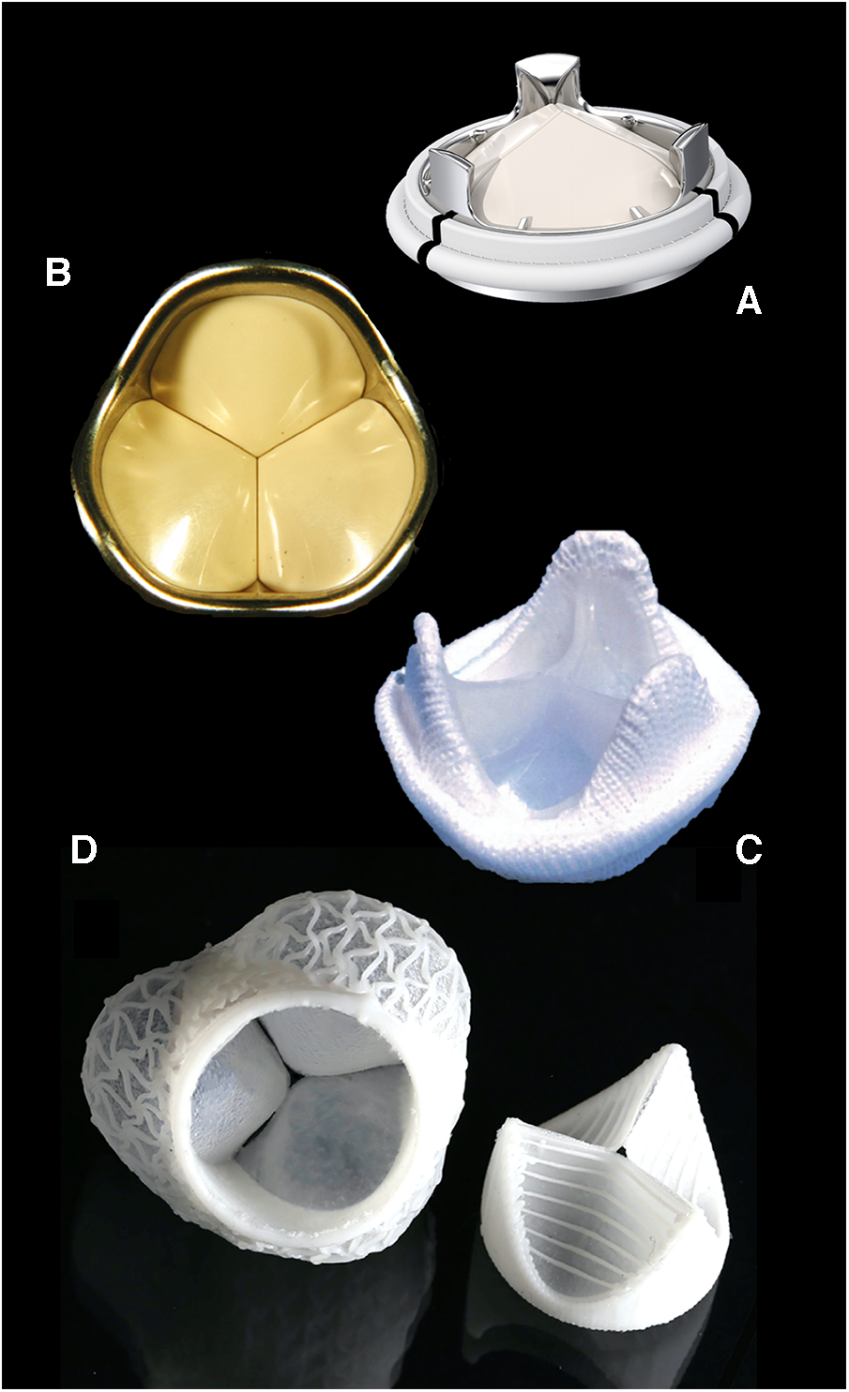
Contemporary valve prostheses catering to elderly patients and optimal anticoagulation controls of industrialised countries are poorly suited for LMICs. Valve designs promising to comply with the high demands of young patients with RHD on leaflet durability and thrombogenicity will rapidly materialise if young patients and poor INR control become the benchmark of valve design. Typical disruptive technologies in-waiting include tri-leaflet mechanical valves: (**A**) Triflo, Novostia, Switzerland (with permission); (**B**) Sievers valve, [from (57) with permission] and polymeric valves; (**C**) Reul–Ghista valve [from (58) with permission]; and (**D**) ETH Zurich valve [from (59) with permission].

As most mechanical heart valves represent designs of the 1970s, little progress has been made towards improved fluid dynamics with better mitigation of platelet activation and clot formation. Yet, some significant innovations have recently emerged. The discovery that high-amplitude flow velocities of short duration close to valve closure cause substantial shear stress with subsequent initiation of the blood coagulation pathways (56) makes disruptive tri-leaflet designs a promising alternative (Figure 2). For tissue valves, on the other hand, the late acknowledgement that remnant immunogenicity plays a crucial role in accelerated bioprosthetic mineralisation (60–62) has validated the use of decellularisation in tissue treatment (54, 63). Yet, clinical trials are scarce, either limited to decellularised homografts (64, 65) or non-cross-linked xenografts (66, 67), with the vast majority of publications failing to assess calcification. However, the fact that the remaining extracellular matrix still exhibited xenogenic antigens (68, 69) further contributed to the revival of the concept of polymer leaflets (70). The first one to pioneer the concept in patients was Foldax®. Soon after successful “first-in-man” implants in aortic valves (70), it was recognised that young patients with RHD present an ideal opportunity for introducing a disruptive technology that could potentially create non-calcifying soft-leaflet valves for life. Should polymer valves prove successful, it will also remove the age barrier for TAVR in patients younger than 60 years in high-income countries.

## Discussion

The reality of anticoagulation control after mechanical heart valve replacement in regions where RHD predominates is not comparable to that in developed countries. Factors such as lower life expectancy due to different underlying pathologies, delayed surgery, and inadequate “compliance” with anticoagulation do not justify the direct uncritical application of Western guidelines. Nonetheless, before radically different heart valve prostheses become available, the choice will continue to be between mechanical and tissue valves.

Our data obtained from 552 consecutive patients who had a mechanical valve replacement for RHD confirm key findings from studies conducted in other LMICs. First, the patients were notoriously under-anticoagulated, although they had carefully been educated by social workers before being discharged into local care regarding the importance of ongoing tests and not falling below a defined INR limit. The rapid decrease in the number of INR tests from 16.1 ± 10.0 to 1.0 ± 3.4 per patient per annum over as short a period as 6 years cannot only be explained by a stabilisation of INR levels over time, as this trend was observed across the board.

Going forward, Thomson–Mangnall's principles (21) provide a starting point for identifying patients at risk for poor adherence: With a significant proportion of patients being on anticoagulation for chronic atrial fibrillation at the time of surgery, an assessment of their compliance history should be possible. If a patient's test records fall outside an acceptable frequency and INR range, tissue valves in combination with non-vitamin K oral anticoagulants have been recommended (71) as a more stable form of anticoagulation, eventually leading to better survival. The rationale behind this is the higher efficacy of anticoagulation in atrial fibrillation than in mechanical heart valves, where higher stroke rates were seen even in industrialised countries despite reasonably good anticoagulation (72, 73).

A travel time of more than an hour to the next INR clinic was highlighted as the second risk factor for safe anticoagulation. This may again pose a challenge since, although rural patients often move to a metropolitan area to increase their likelihood of undergoing valve surgery, a sizeable proportion eventually return to their rural home, particularly male patients at the peak of their productivity who have dependants at their point of origin.

Most importantly, one must individually weigh the risks of poor INR control against a patient's life expectancy. To name a few life-shortening contributors, patients from rural backgrounds and low socio-economic status often present with advanced disease. Similarly, the presence of aortic regurgitation and/or mitral regurgitation also contributes to excess mortality rates.

Crucially, a surgeon working in an LMIC needs to have the self-confidence to be assertive, even if a decision is at variance with what one would normally make under the circumstances prevailing in a developed country. This critical re-assessment may begin at the collective clinical decision level, where differentiated clinical intuition tends to get overruled by chronological age as the primary determinant for valve choice.

On their part, valve companies need to recognise the fact that neither can septua- and octogenarian patients serve as the point of reference for bioprosthetic degeneration, nor are sophisticated self-tests for controlling anticoagulation available. Once young patients from LMICs have been accepted as the challenging new benchmark for valve replacement, exciting new concepts will emerge, leading to better prostheses for all (Figure 2), thus justifying the rollout of such technologies to poor regions with a high burden of RHD in spite of low-profit margins.

## Data Availability

The raw data supporting the conclusions of this article will be made available by the authors without undue reservation.
